# Relationship of Placental Vascular Indices with Macroscopic, Histopathologic, and Intraoperative Blood Loss in Placenta Accreta Spectrum Disorders

**DOI:** 10.1155/2022/2830066

**Published:** 2022-06-24

**Authors:** Mohammad Adya Firmansha Dilmy, Yuditiya Purwosunu, Yudianto Budi Saroyo, Tantri Hellyanti, Noroyono Wibowo, Damar Prasmusinto, Rima Irwinda, Victor Prana Andika Santawi, Hizkia Mangaraja Hasiholan, Rabbania Hiksas

**Affiliations:** ^1^Maternal Fetal Division, Department of Obstetrics and Gynaecology, Faculty of Medicine Universitas Indonesia/Cipto-Mangunkusumo Hospital, Java, Indonesia; ^2^Department of Anatomical Pathology, Faculty of Medicine Universitas Indonesia/Cipto-Mangunkusumo Hospital, Java, Indonesia; ^3^Department of Obstetrics and Gynaecology, Faculty of Medicine Universitas Indonesia/Cipto-Mangunkusumo Hospital, Java, Indonesia

## Abstract

**Introduction:**

Placenta accreta spectrum is an obstetrical complication with a high level of morbidity. The 3-dimensional (3D) power Doppler method has been widely used to improve the diagnosis. Therefore, this study aims to elucidate better the relationship of quantitative placental vascular indices towards macroscopic findings, histopathological grading, and intraoperative blood loss in the disorder.

**Methods:**

A preliminary study using a cross-sectional design was conducted on 34 clinically diagnosed women with PAS. The 3D power Doppler with the VOCAL II software was used to measure the level of vascularization index (VI), flow index (FI), and vascularization flow index (VFI). Gross anatomical appearance and histopathology results were categorized as accreta, increta, and percreta. In addition, the intraoperative blood loss level was measured, and 1500 mL was the cutoff for massive hemorrhage.

**Results:**

The vascularity indexes were VI = 44.2 (23.7–74.9), FI = 35.4 (24.9–57), and VFI = 15.3 (8.5–41.7). The FI value was significant in comparing gross pathological stages (*p*=0.015) and had a moderate positive correlation in relation to blood loss (*r* = 0.449). VI, FI, and VFI above the cutoff values were shown to be strongly associated with blood loss ≥ 1500 cc with aOR 7.00 (95% CI 1.23–39.56), aOR 10.00 (95% CI 1.58–63.09), and aOR 9.16 (95% CI 1.53–54.59), respectively.

**Conclusion:**

This preliminary study demonstrated an initial potential of the FI value from 3D USG power Doppler to predict the depth of PAS invasion before surgery and intraoperative blood loss level.

## 1. Introduction

Placenta accreta spectrum (PAS) is invasive placentation shown by the absence of the decidua basalis layer, mainly due to a deficient Nitabuch's layer, resulting in placental anchoring villi coming deeper towards the myometrium or even beyond [1]. The worldwide incidence of it has continued to increase, along with the rising of a cesarean section rate. A global study has shown that from 1/4.017 cases in 1982, there were 1/272 cases found in 2016 [2]. Particularly, in Indonesia, the incidence of placental invasion was 47.6% in patients with placenta previa and a history of cesarean section [3]. This condition is a potentially life-threatening obstetric complication as it requires inevitable interventions such as manual placental removal, curettage, massive blood transfusion, and hysterectomy [2]. Thus, accurate diagnosis is vital to prepare optimal management. These would allow a prompt referral to a tertiary referral center or placenta accreta management center, accordingly handled by an experienced multidisciplinary perioperative team [4].

Ultrasonography (USG) is considered superior and has a high accuracy diagnostic tool compared to other modalities to early diagnose PAS [5, 6]. The 3-dimensional (3D) power Doppler has been a widely used tool to improve the PAS diagnosis, much better than 2-dimensional (2D) USG [7]. One of the most commonly used parameters is the placenta accreta index (PAI), a qualitative index that estimates the possibility of PAS using USG [8, 9]. However, there are still differences between prenatal ultrasonography, intrasurgical, and histopathological findings. Currently, the relationship between ultrasound findings and the most common complication of PAS surgery, namely, massive bleeding, is still difficult to establish [6].

The uterine and placental vascularizations are crucial for placental development. An abnormal value of placental vasculature has been associated with several obstetrical complications, including PAS [2, 10]. The 3D power Doppler, in combination with the VOCAL II software, allows quantitative evaluation of intraplacental blood circulation, including vascularization index (VI), flow index (FI), and vascularization flow index (VFI) [11]. This vascularization imaging was expected to improve the suitability of prenatal diagnosis with clinical outcomes on PAS. This study explored the relationship between VI, FI, and VFI, measured using 3D USG power Doppler towards anatomic and histologic findings and intraoperative blood loss cases in PAS disorder.

## 2. Methods

### 2.1. Sample Recruitment

This was a preliminary study, using a cross-sectional design. A total of 34 women were recruited in Cipto Mangunkusumo Hospital, Jakarta, from March to December 2021. The hospital is a national referral hospital that receives and treats referred cases from primary, secondary, and tertiary healthcare facilities. All patients clinically diagnosed with the placenta accreta spectrum within those periods were included in this study. In addition, patients' medical records, including USG examination, gross pathological appearance, histopathology results, and the level of blood loss, were collected. Patients with uncomplicated data were excluded from the study.

This study confirmed the principles set out in the World Medical Association Declaration of Helsinki and approved by The Ethical Committee for Research in Humans from The Faculty of Medicine, Universitas Indonesia (KET-1780/UN2. F1/ETIK/PPM.00.02/2021). All of the participants have given their informed consent prior to their inclusion in the study.

### 2.2. Data Collection

The clinical diagnosis of PAS was suspected based on the PAI score. Ultrasound assessment was performed by a maternal-fetal consultant using 3 types of ultrasound machines: Voluson E8, Voluson E6, and Voluson S8 (GE Medical Systems) with the RAB4-8D (4–8 MHz) abdominal volume scanner. The examination was started by performing biometric scans, fetal morphology, and placental localization in 2D for all subjects. This was followed by examining the series of placental bleeding indices (VI, VFI, and FI) using 3D power Doppler with the VOCAL II application method.

The settings for the ultrasound machine used were as follows: pulser repetition frequency (PRF) 0.9 kHz, wall motion filter: low 1, quality: norm, frequency: low, smooth: 4/5, artefact on. The gain values were determined individually based on placenta location, the thickness of the adipose layer, and the scanning conditions affected by those factors. The distance of the probe to the placenta was maintained to remain the same.

The VOCAL II software was used to view the 3D biopsy bleeding, suppress VOCAL, and take a manual shading biopsy with a rotation angle of 30°. These would be followed by displaying a histogram volume that automatically calculates VI, VFI, and FI.

### 2.3. Outcome Measures

The extracted subject's characteristics data include maternal age, gestational age, mode of delivery, gravidity, parity, abortion, previous cesarean section, history of myomectomy or curettage, and types of surgery. The primary outcomes measured include gross pathologic features, histopathological appearance, and the level of blood loss.

### 2.4. Pathologic Examination

The procedure of pathologic examination was done according to tissue pathways for gynecological pathology. The gross anatomy of placenta and uterus was assessed to determine whether there was bulging bluish discoloration. After that, the uterus was put in 10% neutral buffered formalin and formalin fixation for at least 6 days. Seven samples were then biopsied from the suspected abnormal invasion area. The tissues were blocked using paraffin and stained with hematoxylin and eosin (H&E). All histology results were documented based on the villous invasion grades [12]. The decidua was assessed according to the cell morphology, and the depth of villous invasion was classified according to FIGO histological classification [13].

### 2.5. Blood Loss Calculation

Blood loss was calculated intraoperatively using blood volume on the suction jar (amniotic fluid excluded), gauss used, and blood in the drapes and operating field (“Quantification of Blood Loss: AWHONN Practice Brief Number 1,” 2015). The cutoff for massive blood loss was 1500 mL due to occurrence of hemodynamic decompensation after such bleeding [14, 15].

### 2.6. Statistical Analysis

Data were analyzed using Statistical Package for Social Sciences (SPSS) version 25.0 (IBM, United States). The numeric data were first checked for normal distribution using the Shapiro–Wilk test and then presented as mean ± SD if normally distributed and median (min-max) if not normally distributed. For 3 groups of categorical outcomes, the Kruskal–Wallis test was used to identify the significance, as the results showed abnormal data distribution. For 2 groups of categorical outcomes, the unpaired *t*-test or Mann–Whitney test was used. Following that, classification and correlation between numeric variables were analyzed using the Spearman test.

Particularly for blood loss categorical data, the receiver operating characteristic (ROC) curve was used to analyze the corresponding cutoff points, sensitivity, and specificity of each variable. The overall performance of each parameter for predicting massive blood loss (≥1500 cc) was estimated using the area under the curve (AUC). The cutoff points for high overall performance parameters were determined at the point on the curve with the highest value of sensitivity and specificity. Bivariate analysis was performed using the chi-square test, followed by a multivariate association between vascularity indexes variables after adjusting maternal age, gravidity, parity, and abortion status. Each odds ratio (OR) was estimated using 95% confidence interval (95% CI). All results corresponding to *p* values <0.05 (5%) were described as significant and reported.

## 3. Results

### 3.1. Characteristics of Subjects

The median maternal age was 35 (28–41) years old, with 18 patients (52.9%) aged over 35 years old. Most subjects also delivered preterm (<37 weeks), accounted in 26 subjects (76.4%). More than half of subjects also had gravidity greater or equal to 4 (58.8% subjects), parity less or equal to 2 (56% subjects), and no abortion history (70.6%).

The most common type of surgery performed was hysterectomy, underwent in 29 people (85.3%) and the remaining were resection surgery, as many as 5 people (14.7%).  Table 1 provides the characteristics of subjects.

### 3.2. Vascularity Index in Relation to Anatomical Pathologic Features

**Figure 1:**
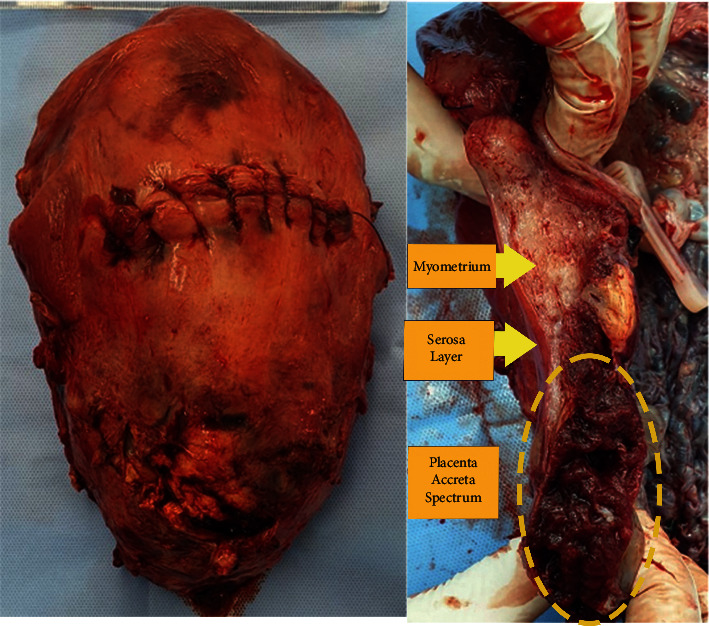
The example of gross pathology results.

The results of this study were VI = 44.2 (23.7–74.9), FI = 35.4 (24.9–57), and VFI = 15.3 (8.5–41.7). By comparing to gross pathological appearance, the FI value was found to be significant (*p*=0.015). The FI value was also higher as PAS grading increased from accreta to increta (Table 2). The example of gross pathology results is shown in Figure 1.

In addition, the histopathological examination revealed 28 increta specimens and 5 percreta specimens, with an unclear result in one specimen. Therefore, only 33 specimens were analyzed.

**Figure 2:**
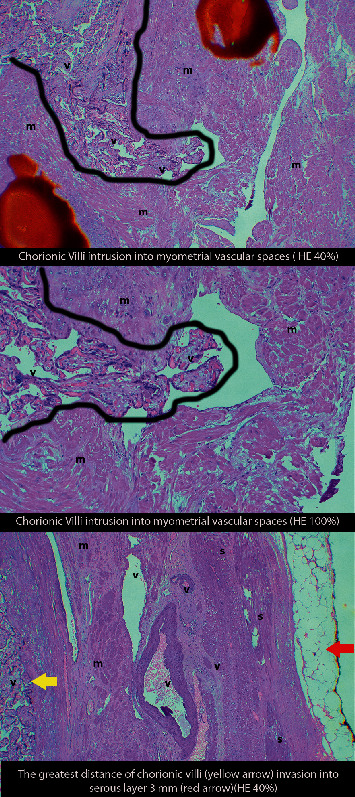
The example of histopathology results. v, chorionic villi; m, myometrium; s, serous layer; black lines, trophoblastic invasion lines.

Although no significance was found among groups, the level of FI was found to increase along with further placental invasion, consistent with the gross pathology result (Table 3). The example of gross pathology results is shown in Figure 2.

### 3.3. Vascularity Index in Relation to Blood Loss

Concerning the blood loss, a moderate positive correlation was found in FI (*r* = 0.449), while other parameters only showed very low to low positive correlation (*r* = 0.126–0.368) (Table 4). To compare, the PAI was also measured. It also showed a significant positive correlation, but the coefficient correlation was still less than FI.

**Figure 3:**
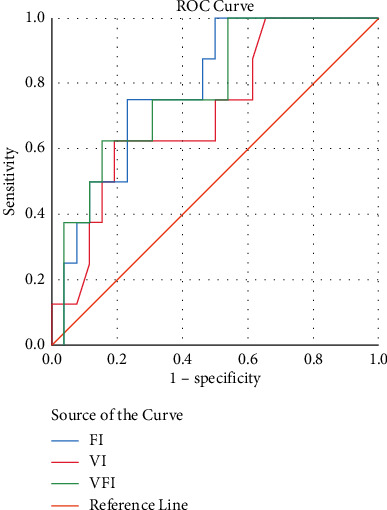
ROC cure of vascularity indexes on massive blood loss.

In predicting massive blood loss >1500 cc, the results showed that the AUC of ROC is acceptable with values ranging 0.71–0.78 (Figure 3). The three indexes for assessing intraplacental vascular circulation have good sensitivity and specificity values related to the diagnostic ability of bleeding >1500 cc on the spectrum of placenta accreta. FI, VI, and VFI values above the cutoff value were strongly associated with blood loss >1500 cc. This value could increase the risk of blood loss relatively high, with OR ranging from 7.00 to 10.00, after being adjusted with maternal age, gravida, parity, and abortion. Even so, the 95% CI has a reasonably high range which is probably due to the small sample size (Table 5).

## 4. Discussion

In this study, the median maternal age was 35 years old. This result was consistent with another study which suggests that advanced maternal age (≥ 35 years old) was a risk factor for PAS development [16]. A study by Carrillo in 2019 also found that women aged over 35 years were associated with the occurrence of PAS, regardless of the cesarean section history, with the risk increasing for each following year by 1.30 times [17]. In addition, we also found that the median for parity and the previous cesarean section was 2. Previous studies have shown that placenta previa with accreta spectrum risk increased in multiparous women and women with a history of cesarean section [18]. The number of cesarean section was associated with the risk of PAS as a previous study showed that three times of cesarean section have increased the risk compared to 2 times with OR 1.28; 95% CI 0.73–2.26 [19].

Related to placental vasculature, there were 3 main vascular indices analyzed: the VI value which estimates the percentage of vascularized tissue, the FI value which represents the average blood velocity of flow, and the VFI value which reflects blood flow and vascularization within the tissue. These vascular indices values are expected to remain constant during the entire normal pregnancy, even in changes in placental volume [11]. Thus, abnormal findings of these values might suggest particular placental pathology.

Our results found a significantly higher median of VI, FI, and VFI compared to previous studies [11, 20]. In contrast, the FI value was lower, although only with slight differences [11, 20]. These findings were consistent with another study that compared these 3 placental vasculatures on morbidly adherent placenta (MAP). They found that VI and VFI values were significantly higher in the MAP group than in normal pregnancy, with almost similar FI values [21]. These data may support the fact that VI and VFI, but not FI, could be good placental vasculature markers for predicting the presence of PAS.

Interestingly, FI values have been found to significantly increase along with further placental invasion in gross pathological features with *p* = 0.015, whereas there was no such finding in 2 other vasculature indices. Lower FI value was found in shallower placental invasion macroscopically. This result was similar to histopathology results, where a higher FI value was found in percreta compared to increta, unlike other indices. The insignificant of microscopic results might be associated with only 2 groups compared, rather than 3 groups like the gross features. The slightly different pathologic results between macroscopic and microscopic findings are possible because the degree of villous adherence is rarely uniform across the placental bed [10]. Also, macroscopic and microscopic pathological results are subjective. In cases of hysterectomy procedure, the vasculature could collapse causing macroscopic changes in radial/arcuate circulation. Histopathological examination was also considered tricky along with the progress of gestational age since the decidual layer may become thinner [10]. Nonetheless, with these results, the FI value may become a potential marker to predict the depth of invasion.

A deeper invasion is associated with higher morbidity, including severe hemorrhage [13]. Here, the FI value showed a significant moderate correlation with the level of blood loss, with a better correlation coefficient than PAI. Since the FI reflects the amount of blood flow, the increase of blood flow was associated with more severe hemorrhage. A previous study has shown that those three vasculature indices were positively correlated with massive bleeding. Though, it was in dilatation and curettage cases [22]. Our results are consistent with another study, which found that intraoperative bleeding was moderately associated with PAS, which could be predicted using 2D and 3D color Doppler [23]. Vascular quantification assessment using 3D Doppler ultrasound proved to be a diagnostic adjunct to identify the possibility of bleeding ≥ 1500 cc. However, the wide range of 95% confidence interval may suggest that other factors might influence the accuracy; thus, additional samples in further studies are required.

This study was limited to a specific USG software and machine. Hence, different settings may reflect different results. Nevertheless, this initial study may become the basis for further research on placental vasculature examination using 3D USG in predicting PAS and its associated morbidities. Therefore, further research is recommended with a broader range of populations to increase its precision.

## 5. Conclusion

In conclusion, this preliminary study demonstrated initial potential of the FI value from 3D USG power Doppler to predict the depth of PAS invasion before surgery and the level of blood loss intraoperatively. Additional diagnostic tests of FI might be particularly useful in referral centers where PAS surgery was performed. Nevertheless, further studies are still required to confirm the result [24].

## Figures and Tables

**Table 1:** Characteristics of subjects.

Variables	*n* (%), *n* = 34	*n* (%), *n* = 34
Maternal age (years old)	35 (28–41)	
< 35		16 (47.1)
≥ 35		18 (52.9)

Gestational age (weeks)	36 (35–39)	
< 37		26 (76.4)
≥ 37		8 (23.6)

Gravidity	4 (2–4)	
< 4		14 (41.2)
≥ 4		20 (58.8)

Parity	2 (1–5)	
≤ 2		19 (56.0)
> 2		13 (44.0)

Abortion	0 (0–3)	
0		24 (70.6)
≥ 1		8 (29.4)

Previous cesarean section	2 (1–4)	
< 2		11 (32.4)
≥ 2		16 (67.6)

History of myomectomy/curettage		
No		24 (70.6)
Yes		10 (29.4)

Types of surgery		
Resection		5 (14.7)
Hysterectomy		29 (85.3)

Blood loss (cc)	1000 (400–2000)	
<1500		26 (76.5)
≥ 1500		8 (23.5)

^*∗*^Data presented as median (min-max).

**Table 2:** Vascularity index on gross pathological appearance

Variables	Gross pathological appearance (*n* = 34)			*P* value
	Accreta (*N* = 10)	Increta (*N* = 16)	Percreta (*N* = 8)	
VI	40.1 (26.4–69.7)	43.0 (23.7–74.9)	54.1 (36.2–73.2)	0.550

FI	33 (24.9–40.6)	35.2 (26.1–44.4)	40.0 (34.0–57.0)	0.015

VFI	12.4 (8.5–28.3)	15.2 (8.7–31.6)	20.9 (12.9–41.7)	0.155

^*∗*^Data presented as median (min-max).

**Table 3:** Vascularity index on histopathological appearance.

Variables	Histopathology appearance (*n* = 33)		*P* value
	Increta (*n* = 28)	Percreta (*n* = 5)	
VI	46.5 (26.4–74.9)	36.2 (23.7–59.9)	0.258
FI	36.2 ± 6.7	38.5 ± 1.7	0.054
VFI	15.4 (8.5–41.7)	14.7 (9.5–22.3)	0.581

^*∗*^Data presented as mean ± SD or median (min-max).

**Table 4:** Correlation of vascular indexes with the level of blood loss.

Variables	The level of blood loss	
	*r*	*P* value
VI	0.126	0.478
FI	0.449	0.008
VFI	0.313	0.072
PAI	0.368	0.032

**Table 5:** ROC curve of vascularity indexes on massive blood loss.

Variables	AUC of ROC	Cutoff	Sensitivity (%)	Specificity (%)	*P* value	Unadjusted OR	Adjusted OR (95% CI)^*∗*^
VI	0.712	≥ 60.4	62.5	80.8	0.031	7.00 (1.238–39.566)	7.00 (1.23–39.56)
FI	0.784	≥ 38.9	75.0	76.9	0.013	10.00 (1.585–63.097)	10.00 (1.58–63.09)
VFI	0.779	≥ 23.2	62.5	84.6	0.017	9.16 (1.539–54.592)	9.16 (1.53–54.59)

^*∗*^Adjusted with maternal age, gravidity, parity, and abortion.

## Data Availability

The datasets analyzed in the study are available from the corresponding author upon request.
